# Rapid and Sensitive Detection of *Vibrio vulnificus* Using CRISPR/Cas12a Combined With a Recombinase-Aided Amplification Assay

**DOI:** 10.3389/fmicb.2021.767315

**Published:** 2021-10-21

**Authors:** Xingxing Xiao, Ziqin Lin, Xianhui Huang, Jinfang Lu, Yan Zhou, Laibao Zheng, Yongliang Lou

**Affiliations:** Wenzhou Key Laboratory of Sanitary Microbiology, Key Laboratory of Laboratory Medicine, Ministry of Education, School of Laboratory Medicine and Life Science, Wenzhou Medical University, Wenzhou, China

**Keywords:** *Vibrio vulnificus*, recombinase-aided amplification assay, CRISPR/Cas12a, early diagnosis, on-site detection

## Abstract

*Vibrio vulnificus* is an important zoonotic and aquatic pathogen and can cause vibriosis in humans and aquatic animals (especially farmed fish and shrimp species). Rapid and sensitive detection methods for *V. vulnificus* are still required to diagnose human vibriosis early and reduce aquaculture losses. Herein, we developed a rapid and sensitive diagnostic method comprising a recombinase-aided amplification (RAA) assay and the CRISPR/Cas12a system (named RAA-CRISPR/Cas12a) to detect *V. vulnificus*. The RAA-CRISPR/Cas12a method allows rapid and sensitive detection of *V. vulnificus* in 40 min without a sophisticated instrument, and the limit of detection is two copies of *V. vulnificus* genomic DNA per reaction. Meanwhile, the method shows satisfactory specificity toward non-target bacteria and high accuracy in the spiked blood, stool, and shrimp samples. Therefore, our proposed rapid and sensitive *V. vulnificus* detection method, RAA-CRISPR/Cas12a, has great potential for early diagnosis of human vibriosis and on-site *V. vulnificus* detection in aquaculture and food safety control.

## Introduction

*Vibrio vulnificus*, a zoonotic and aquatic pathogen found worldwide, causes vibriosis in aquatic animals and humans (Oliver, 2015; Baker-Austin and Oliver, 2018), which can bring heavy economic losses to aquaculture and seriously affect the personal safety of fishermen and consumers, respectively. The fatality rate of human vibriosis caused by foodborne *V. vulnificus* infection is as high as 50%, while it is about 25% if caused by wound infection (Jones and Oliver, 2009). Clinical studies have found that timely treatment after the onset of vibriosis will significantly reduce the mortality of patients, from 100% after 72 h to 33% after 24 h (Klontz, 1988; Heng et al., 2017). However, the key to timely treatment is to detect *V. vulnificus* rapidly and sensitively. Furthermore, to detect *V. vulnificus* in outdoors and resource-poor areas, rapid method without a sophisticated instrument is favored by inspectors (Choi et al., 2017). Therefore, it is important to develop a rapid, sensitive, and unsophisticated method for detection of *V. vulnificus* to better control its spread and permit the early diagnosis of human vibriosis.

The traditional methods for detection of *V. vulnificus* are laborious, time-consuming, and even false positive (O’Hara et al., 2003; Hartnell et al., 2019), which is obviously not suitable for early diagnosis and on-site detection; thus, they are gradually being replaced by simpler and faster nucleic acid amplification technology (NAT) comprising thermocycler-dependent NAT and thermocycler-independent (isothermal) NAT (Asiello and Baeumner, 2011; El Sheikha et al., 2018). In thermocycler-dependent NAT, quantitative PCR (qPCR) assay has been widely used in *V. vulnificus* detection (Campbell and Wright, 2003; Panicker and Bej, 2005). However, qPCR depends on an expensive real-time PCR instrument and well-trained operators, limiting its usage in on-site detection and resource-poor areas. With the development of NAT, isothermal NAT (iNAT)—which does not require sophisticated equipment, is time-saving, and can be carried out under constant temperature conditions—has emerged, such as recombinase-aided amplification (RAA) (Piepenburg et al., 2006; Qi et al., 2019), loop-mediated isothermal amplification (Han and Ge, 2010) and strand displacement amplification (Lu et al., 2017). Based on the advantages mentioned above, iNAT is a very promising method for on-site detection and early diagnosis, especially RAA, which can even be completed within 10 min using body heat (Wang et al., 2017b). Frustratingly, RAA also has some flaws, such as the lower sensitivity compared with qPCR (Moore and Jaykus, 2017; Gallardo et al., 2019) and the relatively complex terminal test involving purification and gel electrophoresis (Daher et al., 2015; Mayboroda et al., 2018).

Recently, a new detection platform based on clustered regularly interspaced short palindromic repeats (CRISPR) and CRISPR-related protein (Cas) called the CRISPR/Cas system has strongly promoted the development of nucleic acid detection technology (Gootenberg et al., 2017; Chen et al., 2018; Li et al., 2018). This platform relies on the collateral cleavage capability of CRISPR RNA (crRNA)-guided Cas12a or Cas13 to ssDNA or ssRNA reporter after recognizing the target nucleic acid (DNA for Cas12a and RNA for Cas13), can satisfy simplicity, speed, and specificity at the same time, and is considered a very promising technology in pathogen detection. Because of the advantages of the CRISPR/Cas system and the DNA-targeting property of Cas12a, CRISPR/Cas12a system shows great potential for the early diagnosis and on-site detection of bacteria and viruses. However, the detection sensitivity of CRISPR/Cas12a alone is very low (Chen et al., 2018; Li et al., 2018). A seminal study by the Doudna lab (Chen et al., 2018) created the DETECTR method, which consists of a recombinase polymerase amplification (RPA) assay and the CRISPR/Cas12a system, and the sensitivity of DETECTR can be as low as the attomolar level. This method not only inherits the advantages of RPA and the CRISPR/Cas12a system, but also avoids the shortcomings of RPA and the CRISPR/Cas12a system. At present, the DETECTR method has been used to detect a variety of pathogens, such as SARS-CoV-2 (Broughton et al., 2020; Wang et al., 2020), *Vibrio parahaemolyticus* (Zhang et al., 2020) and *Pseudomonas aeruginosa* (Mukama et al., 2020).

**FIGURE 1:**
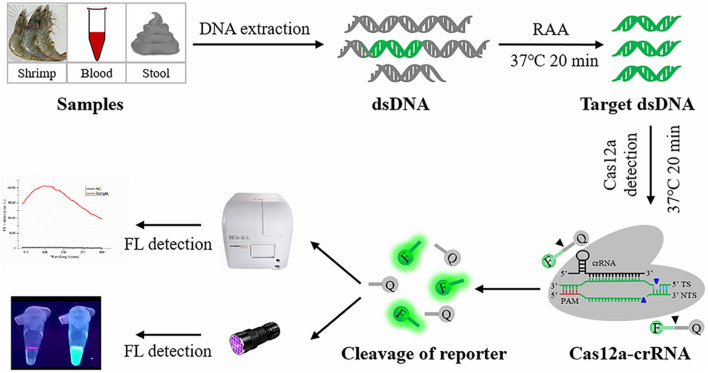
Schematic diagram of RAA-CRISPR/Cas12a assay in the detection of *V. vulnificus*. FL, fluorescence.

In this study, we employed an RAA assay and the CRISPR/Cas12a system to develop a *V. vulnificus* detection method (Figure 1), RAA-CRISPR/Cas12a, targeting the *vvhA* gene. The whole process using this method takes 40 min; the limit of detection is 2 copies/reaction, which is comparable with qPCR; the readout can be evaluated by the naked eye using a UV torch; the fluorescence signal can only be detected in all samples spiked with *V. vulnificus*. The rapid and sensitive characteristics of this method make it a promising candidate for early diagnosis of human vibriosis and on-site *V. vulnificus* detection.

## Materials and Methods

### Bacterial Strains

A total of 10 bacterial strains (five reference strains and five isolation strains) employed in this study were stored in our lab. The five reference strains were *V. vulnificus* (ATCC 27562), *V. harveyi* (ATCC 14126), *V. alginolyticus* (ATCC 17749), *Staphylococcus aureus* (ATCC 25923), and *Bacillus cereus* (ATCC 14579). The five isolation strains were *V. vulnificus*, *V. parahaemolyticus*, *Salmonella typhimurium*, *Edwardsiella piscicida*, and *Aeromonas hydrophila*, which were isolated from eel, clinical sample, clinical sample, carp, and crucian, respectively. All strains were verified by PCR assays targeting the specific segment of 16S rRNA gene.

### Genomic DNA Extraction

Two DNA extraction methods, NaOH-based and Kit-based, were employed to extract bacterial genomic DNA. The NaOH-based method was used to crudely extract the genomic DNA of *V. vulnificus*. Briefly, 50 μL of *V. vulnificus* suspension was added to 200 μL of 0.5 M NaOH solution and incubated at room temperature for 3 min. After being diluted 20-fold with nuclease-free water (Qiagen, Germany), 2 μL of cell lysate was used as template for the RAA assay. A MiniBEST Bacterial Genomic DNA Extraction Kit (TaKaRa, China) was also used to extract bacterial genomic DNA according to the user manual.

### Nucleic Acid Preparation

The *vvhA* gene fragment of *V. vulnificus* (ATCC 27562) obtained by PCR using primer F (5′-CTCTGTTTACCCTTTCTCTTTTAGC-3′) and primer R (5′- GAGTTTGACTTGTTGTAATGTGGGT-3′) was cloned into the pMD19-T vector and then sequenced by Tsingke (Tsingke Biotechnology, China).

Five published vvhA sequences (accession number: M34670.1, KC821520.1, FJ222405.1, AB124802.1, and AB124803.1) were downloaded from GenBank and aligned with the obtained *vvhA* gene sequence using the Clustal Omega^1^. Nine pairs of RAA primers targeting the conserved region of the vvhA sequence were designed according to the Assay Design Manual of the TwistAmp^TM^ DNA Amplification Kits^2^ and were listed in Supplementary Table 1.

As for crRNA design, two factors must be considered: one is that the crRNA sequence lacks overlap with the RAA primers, and the other is that the crRNA sequence targets the conserved region of the RAA amplicon. The ssDNA-FQ reporter modified with fluorophore 6-FAM and quencher BHQ1 (5′-/6-FAM/TTATT/BHQ1/-3′) was used to be *trans-*cleaved by Cas12a and then indicate the presence or absence of the target gene (Chen et al., 2018; Li et al., 2018). crRNA and ssDNA-FQ were synthesized by Sangon Biotech (Shanghai, China), and they were then dissolved in the desired concentration (200 nM for crRNA and 500 nM for ssDNA-FQ) with 1 × NEB buffer 2.1 (NEB ENGLAND BioLabs Inc., United States), aliquoted into 10 μL per tube, and stored at −80°C.

The genomic DNA of *V. vulnificus* (ATCC 27562) extracted by Kit was diluted with 1 × NEB buffer 2.1, and different concentrations (1 × 10^0^ to 1 × 10^8^ copies/μL) of *V. vulnificus* genomic DNA were obtained and stored at −80°C with 6 μL of an aliquot of each gradient.

### RAA-CRISPR/Cas12a Assay

The RAA assay was conducted with an RAA Nucleic Acid Amplification Kit (Jiangsu Qitian Gene Biological Co., China) according to the user manual. Briefly, 25 μL of buffer V, 2 μL of forward primer (10 μM), 2 μL of reverse primer (10 μM), 2 μL of DNA template, 16.5 μL of purified water, and 2.5 μL of magnesium acetate were mixed in tube and then incubated at 37°C for 40 min. The RAA products were analyzed with 2% agarose gel or with the CRISPR/Cas12a system.

A Cas12a-mediated collateral cleavage assay was conducted similarly to the methods used by Chen et al. (2018) and Li et al. (2018). Briefly, 10 μL of 200 nM Cas12a (NEB ENGLAND BioLabs Inc., United States) diluted with 1 × NEB buffer 2.1 was preincubated with 10 μL of 200 nM crRNA for 20 min at 37°C. After this, 10 μL of 500 nM ssDNA-FQ and 2 μL of RAA products were mixed with 20 μL of Cas12a-crRNA complex, and the 32 μL mixture was immediately incubated at 37°C for 35 min. Upon incubation, the readout could be observed using a UV device, such as a UV torch, or detected using a multifunctional microplate reader (λ_ex_: 485 nm and λ_em_: 520 nm). In this study, the RAA reaction time and Cas12a cleavage time were optimized.

### qPCR Assay

A qPCR assay used as a standard method to detect *V. vulnificus* (Campbell and Wright, 2003; Panicker and Bej, 2005) was performed with vvhA-F (5′-TGTTTATGGTGAGAACGGTGACA-3′) and vvhA-R (5′-TTCTTTATCTAGGCCCCAAACTTG-3′) using a CFX96 real-time PCR detection (Bio-Rad, United States) system. The qPCR reaction mixtures contained 10 μL of SYBR^®^ Premix Ex Taq^TM^ II (TaKaRa, China), 0.8 μL of each primer (5 μM), 2 μL of DNA template, and 6.4 μL of nuclease-free water. The reaction condition was: 95°C for 30 s, and 39 cycles of 95°C for 5 s and 60°C for 30 s.

### Detection of Shrimp Samples Using RAA-CRISPR/Cas12a Assay

Eleven fresh shrimps purchased from a local supermarket were proved to be free of *V. vulnificus* by qPCR. Eight of them were spiked with *V. vulnificus* by a researcher according to the methods used by Zhang et al. (2020) and Wang et al. (2017a). Briefly, the fresh shrimps were de-headed and sterilized with 75% ethanol for 2 min. The obtained shrimp samples were incubated in *V. vulnificus* suspensions (1.1 × 10^4^ CFU/mL) for 30 min at 23°C and then transferred onto a clean workbench for bacterial attachment. After 30 min of attachment, 11 shrimps were numbered by this researcher. The RAA-CRISPR/Cas12a assay was then conducted by the other researcher, who did not know the real situation of these shrimps. A Q-tip was used to sample the shrimp by wiping it, and it was then placed into 200 μL of nuclease-free water to obtain *V. vulnificus* suspension. The NaOH-based method mentioned above was performed to extract *V. vulnificus* genomic DNA, and the Kit-based extraction method was used as a comparative test. 2 μL of genomic DNA extracted by these two methods was used as template for the RAA-CRISPR/Cas12a assay.

### Detection of Human Blood and Stool Samples Using RAA-CRISPR/Cas12a Assay

Blood and stool samples were collected from three healthy volunteers, and 100 μL of blood or 200 mg of stool was added into the tube containing 1.1 × 10^3^ CFU of *V. vulnificus*. Then, these blood and stool samples were used to extract genomic DNA using the MiniBEST Universal Genomic DNA Extraction Kit Ver.5.0 (TaKaRa, China) and the TIANamp Stool DNA Kit (TIANGEN, China), respectively. 2 μL of genomic DNA extracted from spiked blood and stool samples were then used as templates for RAA-CRISPR/Cas12a assay, while 2 μL of blood or stool DNA was used as a negative control.

## Results

### Screening an Optimal Primer Set for RAA Assay

**FIGURE 2:**
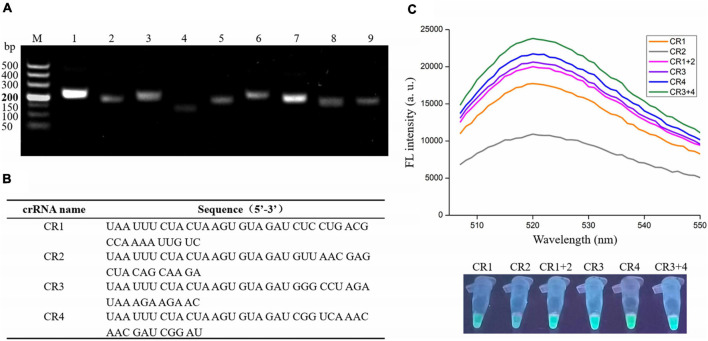
Screening optimal RAA primers and crRNA for the RAA-CRISPR/Cas12a assay. **(A)** Gel electrophoresis analysis of RAA products amplified with different primer set. M, 500 DNA marker; lanes 1–9, RAA products amplified by primer set 1, 2, 3, 4, 5, 6, 7, 8, and 9, respectively. **(B)** crRNA sequences designed in this study. **(C)** Analysis of fluorescence signals triggered by the different crRNA using a multifunctional microplate reader (upper) or a UV torch (below). Data is one representative of three experiments.

To obtain the optimal primers, nine primer sets were designed (Supplementary Table 1), and the RAA assay was performed with *V. vulnificus* genomic DNA and each primer set. The primers were then screened according to the gel electrophoresis of RAA products. As shown in Figure 2A, the predicted bands of each RAA product were visible; however, the intensity of two bands amplified with the No. 1 primer set (F1: 5′-TTCAACGCCACACGAGACTGGTGTAATGCGG-3′ and R1: 5′- CCAATGTAAGTGCGGCGGTTTGCCCAACTCTGG-3′) and the No. 7 primer set (F7: 5′- TTATGGTGAGA ACGGTGACAAAACGGTTGCGGG-3′ and R7: 5′- CCTTCC CAATACCATTTCTGTGCTAAGTTCGC-3′) were significantly stronger than the other seven bands, indicating the high amplification efficiency of the No. 1 and No. 7 primer sets. Therefore, these two primer sets were selected as candidates for subsequent RAA assay, and their amplicons were used to design crRNA.

### Screening an Optimal crRNA for RAA-CRISPR/Cas12a Assay

According to the two factors mentioned in Materials and Methods, only four crRNAs (CR1 and CR2 targeting the No. 1 amplicon; CR3 and CR4 targeting the No. 7 amplicon) were designed (Figure 2B). Because the efficiency of each crRNA and crRNAmix (crRNA mixture) in triggering the *trans-*cleavage capability of Cas12a may be different (Creutzburg et al., 2020; Wang et al., 2020), the RAA-CRISPR/Cas12a assay was performed using *V. vulnificus* genomic DNA as template and F1/R1 or F7/R7 as primer set to test the capacity of CR1, CR2, CR1 + 2, CR3, CR4, and CR3 + 4 and then screen an optimal crRNA. As shown in Figure 2C, all four crRNAs and the two crRNAmixs could trigger fluorescence signal generation; however, the fluorescence signals triggered by crRNAmixs were stronger than those triggered by single crRNA. Furthermore, CR3 + 4 triggered a stronger fluorescence signal than CR1 + 2. Therefore, CR3 + 4 and its corresponding primer set, F7/R7, were chosen as the optimal crRNA and primer set, and would be used in the subsequent RAA-CRISPR/Cas12a assay.

### Optimizing RAA Reaction Time and Cas12a Cleavage Time

**FIGURE 3:**
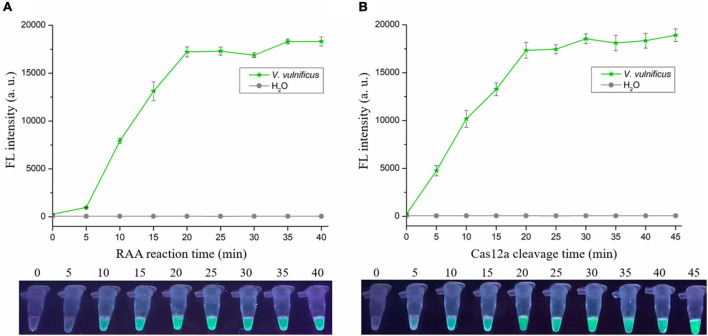
Optimizing the RAA reaction time and Cas12a cleavage time. The RAA-CRISPR/Cas12a assay was performed using 1 × 10^4^ copies/μL of *V. vulnificus* genomic DNA as the template, F7/R7 as the primer set, and CR3 + 4 as the crRNA to optimize the RAA reaction time **(A)** and Cas12a cleavage time **(B)**, and the fluorescence signal was analyzed using a multifunctional microplate reader (upper) or a UV torch (below).

To shorten the assay time with minimal difference in reaction efficacy, we optimized the RAA reaction time and Cas12a cleavage time using the RAA-CRISPR/Cas12a assay with the same template concentration of *V. vulnificus* genomic DNA (1 × 10^4^ copies/μL). As for optimization of RAA reaction time, 0, 5, 10, 15, 20, 25, 30, 35, and 40 min were tested. The results showed that fluorescence intensity reached a plateau after 20 min (Figure 3A), indicating that 20 min was the optimal time for RAA reaction. As for optimization of Cas12a cleavage time, 0, 5, 10, 15, 20, 25, 30, 35, 40, and 45 min were tested. The results shown in Figure 3B indicated that 20 min was the optimal time for Cas12a cleavage. Therefore, the reaction time for the RAA-CRISPR/Cas12a assay we developed to detect *V. vulnificus* was 40 min, consisting of 20 min for the RAA reaction and 20 min for Cas12a cleavage.

### Sensitivity of RAA-CRISPR/Cas12a Assay in the Detection of *Vibrio vulnificus*

**FIGURE 4:**
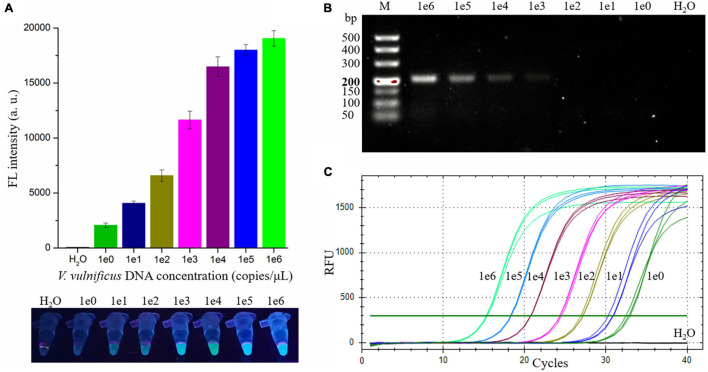
Evaluating the sensitivity of the RAA-CRISPR/Cas12a, RAA, and qPCR assay in *V. vulnificus* detection. 2 μL of nuclease-free water and 1 × 10^0^ to 1 × 10^6^ copies/μL of *V. vulnificus* genomic DNA were used as templates in these assays. **(A)** The sensitivity of the RAA-CRISPR/Cas12a assay. The results were detected using a multifunctional microplate reader (upper) or a UV torch (below). **(B)** The sensitivity of the RAA assay. RAA reaction time was 20 min, consistent with the RAA-CRISPR/Cas12a assay. The results were analyzed using gel electrophoresis. **(C)** The sensitivity of the qPCR assay. qPCR was conducted with the CFX96 real-time PCR detection systems, and the amplification curves of each sample were shown in this figure. Data is one representative of three experiments.

To evaluate the sensitivity of the RAA-CRISPR/Cas12a assay in detecting *V. vulnificus*, 2 μL of different concentrations (1 × 10^0^ to 1 × 10^6^ copies/μL) of *V. vulnificus* genomic DNA and nuclease-free H_2_O were used as RAA templates, and 2 μL of RAA product was then detected with a Cas12a-mediated cleavage assay. As shown in Figure 4A, all samples except H_2_O could generate fluorescence signals detected by a multifunctional microplate reader or a UV device, indicating that the limit of detection (LOD) of this method in *V. vulnificus* detection reached 2 copies/reaction.

To compare the sensitivity of RAA-CRISPR/Cas12a with RAA, qPCR, or CRISPR-Cas12a in the detection of *V. vulnificus*, we also assessed the sensitivity of the RAA, qPCR, and CRISPR-Cas12a assay. As for the sensitivity of the RAA assay that was performed under the same condition as the RAA-CRISPR/Cas12a assay, the result of gel electrophoresis employed to analyze the RAA products showed that the LOD of the RAA assay was 1 × 10^3^ copies/μL (Figure 4B), which was lower than the sensitivity of RAA-CRISPR/Cas12a. The LOD of qPCR assay was 2 copies/reaction (Figure 4C), consistent with the sensitivity of RAA-CRISPR/Cas12a assay. As for the CRISPR-Cas12a assay, we did not detect a fluorescence signal from all samples, even though the sample concentration was 1 × 10^8^ copies/μL (data not shown), which was consistent with the reports that the detection sensitivity of CRISPR-Cas12a alone was very low (Chen et al., 2018; Li et al., 2018).

Taken together, the sensitivity of the RAA-CRISPR/Cas12a assay we established was two copies of *V. vulnificus* genomic DNA per reaction, which is comparable with qPCR but significantly higher than that of RAA and CRISPR-Cas12a.

### Specificity of RAA-CRISPR/Cas12a Assay in Detecting *Vibrio vulnificus*

**FIGURE 5:**
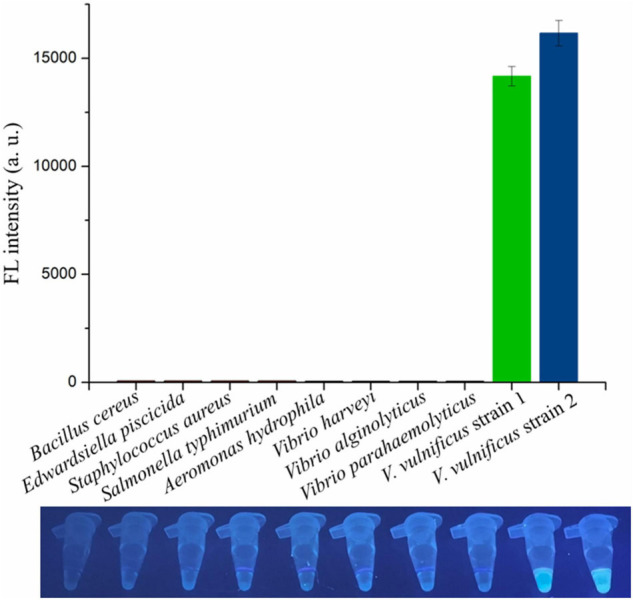
Evaluating the specificity of RAA-CRISPR/Cas12a assay in *V. vulnificus* detection. Ten bacterial strains were used to evaluate the specificity of RAA-CRISPR/Cas12a assay, and the fluorescence sigils were analyzed using a multifunctional microplate reader (upper) or a UV torch (below). *V. vulnificus* strain 1 was isolated from eel, and strain 2 was isolated from human.

The genomic DNA extracted from two *V. vulnificus* strains and eight other strains of foodborne pathogenic bacteria were used to assess the specificity of the RAA-CRISPR/Cas12a assay in *V. vulnificus* detection. The results showed that the fluorescence signal could be detected in those two *V. vulnificus* strains using this method, but not in the strains of *Bacillus cereus*, *Edwardsiella piscicida*, *Staphylococcus aureus*, *Salmonella typhimurium*, *Aeromonas hydrophila, V. harveyi*, *V. alginolyticus*, and *V. parahaemolyticus* (Figure 5), indicating no cross-reactions of the RAA-CRISPR/Cas12a assay in the detection of *V. vulnificus*. Therefore, the method we established displayed a high specificity for *V. vulnificus* detection.

### Detection of *Vibrio vulnificus* in Spiked Samples With RAA-CRISPR/Cas12a Assay

**FIGURE 6:**
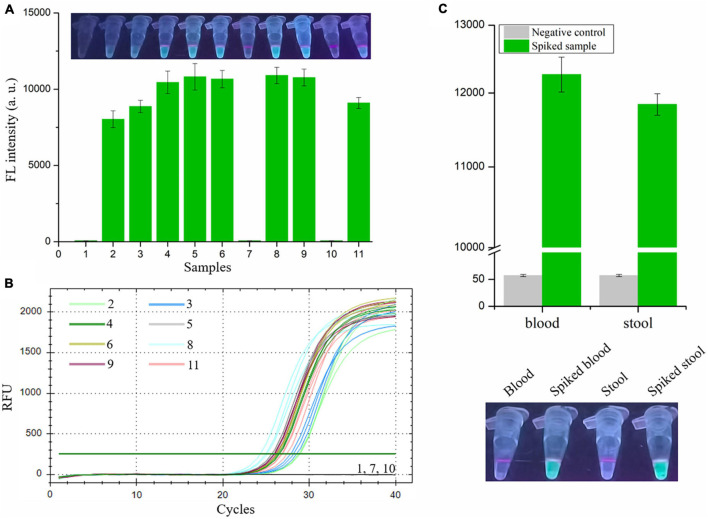
Analysis the feasibility of RAA-CRISPR/Cas12a assay in the detection of *V. vulnificus* in spiked samples. The genomic DNA was extracted from 11 shrimps, eight of which were spiked with 1.1 × 10^4^ CFU/mL of *V. vulnificus*, using the NaOH-based DNA extraction method. **(A)** The RAA-CRISPR/Cas12a assay was performed to detect *V. vulnificus* in those 11 DNA samples using a multifunctional microplate reader (below) or a UV torch (upper). **(B)** The qPCR assay was performed as a standard method to detect *V. vulnificus* in those 11 DNA samples. The amplification curves of each sample were shown. Data is one representative of three experiments. **(C)** Human blood and stool samples were employed to evaluate the feasibility of RAA-CRISPR/Cas12a assay in diagnosis of human vibriosis. 100 μL of blood or 200 mg of stool was added into the tube containing 1.1 × 10^3^ CFU of *V. vulnificus*, and then these samples were used to extract genomic DNA. 2 μL of genomic DNA extracted from spiked samples were used as templates for RAA-CRISPR/Cas12a assays, while 2 μL of blood DNA or stool DNA was used as a negative control. Fluorescence signals were analyzed using a multifunctional microplate reader (upper) or a UV torch (below). Data is one representative of three experiments.

Finally, we evaluated the performance of the RAA-CRISPR/Cas12a assay in the detection of shrimp samples, drawing on eight *V. vulnificus*-spiked samples and three *V. vulnificus*-free samples. This experiment was conducted by two researchers: one was responsible for preparation of the 1.1 × 10^4^ CFU/mL *V. vulnificus*-spiked samples and numbered the 11 shrimps, while the other one with no idea about the situation of shrimps extracted the genomic DNA from shrimps using the NaOH-based and Kit-based methods and then carried out the RAA-CRISPR/Cas12a assay. As shown in Figure 6A, the fluorescence signal could only be detected in eight spiked samples using the RAA-CRISPR/Cas12a assay, which was exactly matched with the results of the qPCR assay (Figure 6B), indicating the high accuracy of this method in *V. vulnificus* detection. Moreover, the RAA-CRISPR/Cas12a assay was also performed using the DNA template extracted through the Kit-based method, which still only detected all the spiked samples (Supplementary Figure 1).

To investigate whether our proposed RAA-CRISPR/Cas12a method has potential to diagnose human vibriosis using human blood or stool samples, the blood and stool samples spiked with 1.1 × 10^3^ CFU of *V. vulnificus* were prepared and used to extract genomic DNA. 2 μL of genomic DNA extracted from blood or stool samples were used as templates. As shown in Figure 6C, apart from the negative controls, the fluorescence signal could be detected in all spiked samples. These results indicated that the RAA-CRISPR/Cas12a assay could resist the influence of human genomic DNA and gut flora DNA, further implying the great feasibility of this assay in the detection of human samples.

Taken together, these results showed that the presented RAA-CRISPR/Cas12a assay could be used to detect *V. vulnificus* in the samples collected from seafood and human beings.

## Discussion

*Vibrio vulnificus* is a mesophilic and zoonotic bacterium (Oliver, 2015; Baker-Austin and Oliver, 2018). With global warming, the populations of *V. vulnificus* are larger, and cases of vibriosis are increasing (Baker-Austin et al., 2013, 2018), seriously threatening aquaculture, food safety, and human health. A more rapid and sensitive detection method is good for reducing the harm caused by *V. vulnificus* infection. Currently, the reported methods for *V. vulnificus* detection are based on NAT and can be divided into two main types: qPCR-based method (Campbell and Wright, 2003; Panicker and Bej, 2005) and iNAT-based method (Table 1), which mainly depends on a real-time PCR instrument or lateral flow dipstick (Han and Ge, 2010; Surasilp et al., 2011; Yang et al., 2020, 2021). However, the qPCR-based and iNAT-based pathogen detection methods depending on the real-time PCR instrument are not convenient for use in on-site detection and resource-poor areas, while the iNAT-based pathogen detection methods depending on the lateral flow dipstick or gel electrophoresis analysis show lower sensitivity than qPCR (Panicker and Bej, 2005; Surasilp et al., 2011; Wang et al., 2017b; Gallardo et al., 2019). To circumvent these defects, we developed an RAA-CRISPR/Cas12a assay to detect *V. vulnificus* (Figure 1), which does not require a sophisticated instrument, only takes 40 min from adding DNA templates to obtaining the results (Figure 3), and can detect *V. vulnificus* genomic DNA in as low as 2 copies/reaction (Figure 4A).

**TABLE 1:** Comparison of different methods for detection of *Vibrio vulnificus*.

**Method**	**Equipment required**	**Speed**	**Sensitivity**	**Specificity**	**References**
Culture	Incubator	Days	Variable	Case-specific	O’Hara et al., 2003; Hartnell et al., 2019
qPCR	Real-time PCR instrument	Hours	High	High	Campbell and Wright, 2003; Panicker and Bej, 2005
iNAT^#^	Real-time PCR instrument	Minutes	High	High	Han and Ge, 2010; Yang et al., 2020
	LFD* or electrophoresis apparatus	Minutes, hours	Medium	High	Surasilp et al., 2011; Yang et al., 2021
RAA-CRISPR/Cas12a	UV torch	Minutes	High	High	This study

*^#^Isothermal nucleic acid amplification technology.*

*Lateral flow dipstick.

The target gene of the presented method is *vvhA*, which encodes an important toxin hemolysin in *V. vulnificus* pathogenicity (Kreger and Lockwood, 1981; Lee et al., 2004). Because of the species specificity and high conservation of *vvhA* gene (Wright et al., 1985; Morris et al., 1987; Hill et al., 1991), detection of the *vvhA* gene has been used as a standard method to identify *V. vulnificus* (Hill et al., 1991; Campbell and Wright, 2003). Because of the diversity of *vvhA* gene (Senoh et al., 2005), six vvhA sequences were aligned, and the conserved region was then used to design the RAA primers and crRNA sequences. The specificity tests showed that only two *V. vulnificus* strains (a clinical isolate and an eel isolate) could be detected using the RAA-CRISPR/Cas12a method (Figure 5), indicating that the primers and crRNA sequences we designed were valid. Moreover, we also evaluated the feasibility of this method in the detection of spiked samples. As for detection of *V. vulnificus* in shrimps (Figure 6A), the results showed that the RAA-CRISPR/Cas12a assay could detect all the spiked samples, indicating the great potential of this assay for on-site *V. vulnificus* detection.

Apart from detection of *V. vulnificus* in spiked shrimp samples using the RAA-CRISPR/Cas12a method, we also investigated the feasibility of this method in diagnosis of human vibriosis. As we all known, primary septicemia and gastroenteritis are two major clinical syndromes of *V. vulnificus* infections (Chuang et al., 1992; Shapiro et al., 1998). Therefore, human blood and stool samples were used to conduct this experiment (Figure 6C). The results demonstrated that our proposed RAA-CRISPR/Cas12a method showed high accuracy in the detection of human samples, indicating the great potential of this method for the early diagnosis of human vibriosis.

In conclusion, our presented method, the RAA-CRISPR/Cas12a assay, simultaneously satisfies speed, specificity, sensitivity, and unsophisticated to detect *V. vulnificus*, and shows great potential for on-site *V. vulnificus* detection in aquaculture and food safety and for the early diagnosis of human vibriosis, especially in resource-poor areas.

## Data Availability Statement

The original contributions presented in the study are included in the article/Supplementary Material, further inquiries can be directed to the corresponding author/s.

## Author Contributions

XX, LZ, and YL designed the study. XX and ZL wrote the manuscript. ZL and XH performed the experiments and analyzed the data. JL and YZ reviewed the manuscript. All authors contributed to the article and approved the submitted version.

## Conflict of Interest

The authors declare that the research was conducted in the absence of any commercial or financial relationships that could be construed as a potential conflict of interest.

## Publisher’s Note

All claims expressed in this article are solely those of the authors and do not necessarily represent those of their affiliated organizations, or those of the publisher, the editors and the reviewers. Any product that may be evaluated in this article, or claim that may be made by its manufacturer, is not guaranteed or endorsed by the publisher.

## Footnotes

1
https://www.ebi.ac.uk/Tools/msa/clustalo/

2
https://www.twistdx.co.uk
