# Supplementary material for: Rapid and Sensitive Detection of Vibrio vulnificus Using CRISPR/Cas12a Combined With a Recombinase-Aided Amplification Assay
Source: Front Microbiol. 2021 Oct 21;12:767315. doi: 10.3389/fmicb.2021.767315 (PMC8566878; doi:10.3389/fmicb.2021.767315)
Supplement: Supplementary file 1 [file Table_1.DOC]

**Table S1** | RAA primers designing in this study.

| Primers | Sequences（5’—3’） | Product size（bp） |
| --- | --- | --- |
| RAA-F1 | TTCAACGCCACACGAGACTGGTGTAATGCGG | 233 |
| RAA-R1 | CCAATGTAAGTGCGGCGGTTTGCCCAACTCTGG |
| RAA-F2 | GCTTCCATCGATGTTCGCGTCAATGTGGCAC | 199 |
| RAA-R2 | CAATGTAAGTGCGGCGGTTTGCCCAACTCTGG |
| RAA-F3 | CCTTGGTGTTTGATACAAAAGACTATCGCATC | 216 |
| RAA-R3 | CGTCATAGTTCGGTTTGAAGTTGGAATAAGAG |
| RAA-F4 | CTGGAGCTGTCACGGCAGTTGGAACCAAGT | 140 |
| RAA-R4 | CTAAGTTCGCACCACACTGTTCGACTGTGAGC |
| RAA-F5 | ACGCTCACAGTCGAACAGTGTGGTGCGAAC | 190 |
| RAA-R5 | CTTGTTGTAATGTGGGTTTCCAACGCGCCTG |
| RAA-F6 | TACTTACGGTTACTCCATCGGTATTAACGG | 234 |
| RAA-R6 | CATCCAAGCTCTTGGCGGCGCAGTTCATCAC |
| RAA-F7 | TTATGGTGAGAACGGTGACAAAACGGTTGCGGG | 210 |
| RAA-R7 | CCTTCCCAATACCATTTCTGTGCTAAGTTCGC |
| RAA-F8 | TTCGGTTAACGGCTGGAGCTGTCACGGCAG | 181 |
| RAA-R8 | CTTATCGCCTTCCCAATACCATTTCTGTGC |
| RAA-F9 | CGCTCACAGTCGAACAGTGTGGTGCGAACTTAGC | 189 |
| RAA-R9 | CTTGTTGTAATGTGGGTTTCCAACGCGCCTG |

**
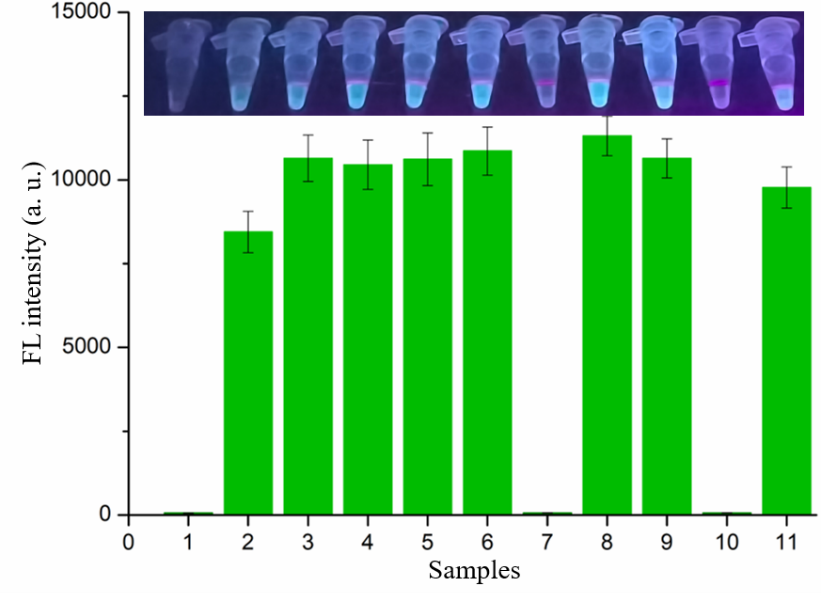
**

**Figure S1 |** Analysis of RAA-CRISPR/Cas12a assay in the detection of *V. vulnificus* in spiked shrimp sampses. Genomic DNA was extracted from 11 shrimps, 8 of which were spiked with 1.1 × 10^4^ CFU/mL *V. vulnificus*, using Kit-based DNA extraction method. Fluorescence signal could be detected in all spiked samples using a multifunctional microplate reader (below) or a UV torch (upper).
